# Primary cutaneous ALK-negative anaplastic large cell lymphoma

**DOI:** 10.1093/omcr/omae177

**Published:** 2025-10-29

**Authors:** Pratiksha Mishra, Chandraprava Mishra, Siddhartha Dash

**Affiliations:** Department of Pathology, S.C.B. Medical College & Hospital, Mangalabag, Cuttack, Odisha, 753007, India; Department of Pathology, S.C.B. Medical College & Hospital, Mangalabag, Cuttack, Odisha, 753007, India; Department of Dermatology, and Venereology, S.C.B. Medical College & Hospital, Mangalabag, Cuttack, Odisha, 753007, India

**Keywords:** anaplastic, cutaneous, large cell lymphoma

## Case description

**Figure 1:**
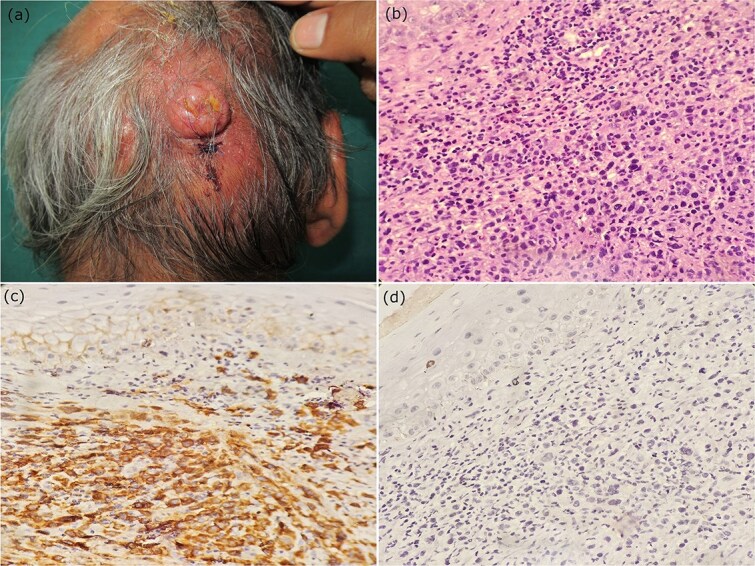
(a) Two well circumscribed nodules present over an erythematous, indurated plaque on scalp (b) histopathology revealed dense dermal infiltrates of round to oval, medium to large sized pleomorphic cells with abundant amount of eosinophilic cytoplasm and irregular shaped nuclei with some showing prominent nucleoli (H&E, X400). (c,d) Immunohistochemistry showing CD 30+ and ALK- tumor cells (IHC, X400).

A 61-year-old male presented with two swelling over scalp for three months. There was no history of constitutional symptoms. General examination was unremarkable. On cutaneous examination, two well circumscribed nodule of size 4 × 3 cm and 1 × 1 cm were present over an erythematous, indurated plaque. There was ulceration over the surface of the larger nodule. (Fig. 1a). Dermoscopy (Dermlite DL4, 10X magnification) revealed reddish-pink background, ulceration, arborizing vessels, scale, and yellow crust.

## What is the diagnosis?

Biochemical, hematological, and radiological parameters were within normal limits. Histopathology revealed dense dermal infiltrates with no involvement of epidermis. Individual tumor cells were round to oval, medium to large sized pleomorphic cells with abundant amount of eosinophilic cytoplasm and irregular horseshoe-shaped nuclei with some showing prominent nucleoli (Fig. 1b). Immunohistochemistry revealed strong and diffuse positive for CD30, and positive for CD3, CD45, but negative for ALK, CD15, CD20. A final diagnosis of primary cutaneous ALK-negative large cell lymphoma (PC-ALCL) was made based on clinical, histopathology and immunohistochemistry criteria. Patient was referred to hematology, where the patient was started on cyclophosphamide, adriamycin, vincristine, and prednisolone (CHOP) regimen.

## Discussion

Cutaneous T-cell lymphomas (CTCL) represents a form of non-Hodgkin’s lymphoma which accounts for 65% to 75% of all cutaneous lymphomas [1]. CD-30+ cutaneous lymphoproliferative disorders account for 30% of all CTCL, and includes lymphomatoid papulosis, primary cutaneous anaplastic large cell lymphoma (PC-ALCL), and borderline lesions, and constitute the second most common group of CTCL after mycosis fungoides and Sezary syndrome [1]. PC-ALCL is a primary cutaneous CD30-positive lymphoproliferative disease of the skin that usually occurs in middle-aged and elderly people [2]. This is a rare lymphoma and the exact incidence is unknown. In contrast to systemic ALCL, PC-ALCL lacks expression of anaplastic lymphoma kinase (ALK) gene. PC-ALCL is prevalent on the trunk, face, and limbs, presents as either isolated or limited reddish-brown papules, nodules, or swellings on human skin. In advanced stages, it mostly presents as ulcerative lesions, with central necrosis and disc-like elevated margins. Extracutaneous dissemination occurs in about 10% of cases of PC-ALCL, which mainly manifests as regional lymph node involvement [3]. The mean duration of extracutaneous involvement is 18 months, which includes dissemination to regional/extensive nodal involvement or visceral involvement [4]. Oliveira et al. described a case of apical lung involvement after seven years of cutaneous ALCL [5]. Lymphomatoid papulosis is histologically and immunophenotypically similar to PCALCL, however, clinically it is characterized by appearance of smaller (< 1 cm) papules and nodules with a waxing and waning course [2]. Mycosis fungoides with large cell transformation (MF-LCT) occurs in long standing classic lesions of mycosis fungoides with development of tumor nodules, and histopathologically characterized by epidermotropic and dermal aggregates of CD30+ tumor cells, which occurs less commonly in PCALCL [2]. Systemic ALCL (S-ALCL) with secondary skin involvement is differentiated from PCALCL by means of ALK gene rearrangements, but ALK negative S-ALCL is differentiated on the basis of clinical presentation, prior history and EMA positivity [1]. Localized lesions can be treated with surgical excision and radiation, but carries a poor prognosis. This case is being presented because of its rarity and the need for its early diagnosis and management, which could prevent its systemic dissemination.
